# Climate change poses a threat to nutrition and food security in Kilifi County, Kenya

**DOI:** 10.4102/phcfm.v14i1.3718

**Published:** 2022-10-31

**Authors:** Susan J. Cheruiyot, Mary Kimanthi, Jacob S. Shabani, Nelson F. Nyamu, Catherine Gathu, Felix Agoi, Fleur De Meijer

**Affiliations:** 1 Department of Family Medicine, Faculty of Health Sciences, The Aga Khan University Hospital, Nairobi, Kenya; 2 Department of Population Health, Faculty of Health Sciences, The Aga Khan University Hospital, Nairobi, Kenya

**Keywords:** climate change, nutrition, food security, communities’ experience, Kenya

## Abstract

Over the last decades, increased emission of greenhouse gases has led to hot weather extremes, heavy precipitation and worsening of agricultural and ecological droughts. Although Africa’s contribution to climate change is minimal, the continent is especially vulnerable to its effects. This report aims to describe the effect of climate change leading to drought in Kilifi County, Kenya, and the communities’ experiences of this effect on food availability. During their community rotation, residents from a university in Nairobi, Kenya, evaluated changes in weather patterns and nutrition indicators in Kilifi County and conducted focus group discussions (FGDs) with community members and health care stakeholders to explore challenges in access to adequate nutrition and possible local solutions. Kilifi County has one of the highest rates of undernutrition in Kenya, with one in five under-5 children being underweight. County data showed that rainfall in the last 4 years has become increasingly unpredictable, resulting in reduced household milk production, one of the indicators of nutrition security. Three major themes emerged from the FGDs: lack of food variety, collapse of drought mitigating projects and increasing poverty levels. Possible solutions to these problems include promoting alternatives to the current diet that are culturally sensitive and adaptable to recent climate changes, ensuring continuity of agricultural and financial support projects and improved local leadership and governance.

## Background

Climate change is a rising global concern, and taking urgent action against it is one of the United Nations (UN’s) Sustainable Development Goals.^1^ Despite Africa’s minimal contribution to greenhouse gas emissions, the continent is unprotected from the effects of climate change. This has resulted in reduced agricultural yield, food insecurity, a rise in poverty levels and reduced human productivity related to health and farming opportunities.^2,3,4,5^ A postgraduate department at a university in Nairobi, Kenya, has, since 2012, undertaken community diagnosis and intervention projects in Kilifi County, a coastal region of Kenya, as part of its Community Oriented Primary Care (COPC) programme. This report aims to describe the effect of climate change on experienced drought in Kilifi County, Kenya, and the communities’ experiences of this effect on food availability.

## Study location

Kilifi is one of 47 counties in Kenya. It is located in the south-eastern part of Kenya and bounded easterly by the Indian Ocean.^6^ Tsangatsini, the area of study, is a vast region in Kilifi County with a population density of 81 people per square metre. Poverty levels are high in this area: 80% of residents perform casual work to make ends meet, and in most households, the husband is the sole income provider.^7^

## Methodology

To evaluate changes in weather patterns and the impact of climate change on nutrition indicators, county-specific quantitative data were collected by residents. Household milk production is used as an index for drought and is associated with household food and nutrition security.^8,9^ Focus group discussions (FGDs) were also conducted with different community members and community health care stakeholders to explore the challenges in access to adequate nutrition and possible local solutions. Eight FGDs with a total of 71 participants were conducted: five with community members (FGD 1–5), two with community health volunteers (CHVs) (FGD 6–7) and one with health care workers (HCWs) (FGD 8).

## Changes in Kilifi rainfall patterns and livestock milk production

**FIGURE 1:**
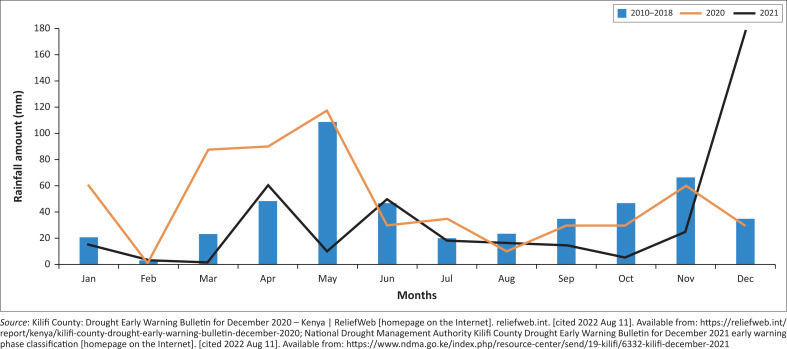
Average rainfall in Kilifi County (mm per month).

Kilifi County experiences two annual rainfall peaks: the short rains in March – May and the long rains in August–October.^6,10,11^ Between 2010 and 2018, these two peaks have occurred regularly, allowing for proper timing of planting and harvesting with fairly good yield.^12,13^ In 2020, unpredictable rainfall patterns were witnessed, with heavy rainfall in the short rains period ruining the burgeoning crops, and a later drier period in August when the heavy rains were expected (see Figure 1). The year 2021 was characterised by minimal rainfall between March and May, a lower-than-expected downpour during the long rains period and heavy rains with flash floods in December (see Figure 1).^11,14^

This led to a drought alarm being issued by the Kenya National Drought Management Authority in March 2022, and food relief was distributed to offset the anticipated rise in food insecurity.^15^

**FIGURE 2:**
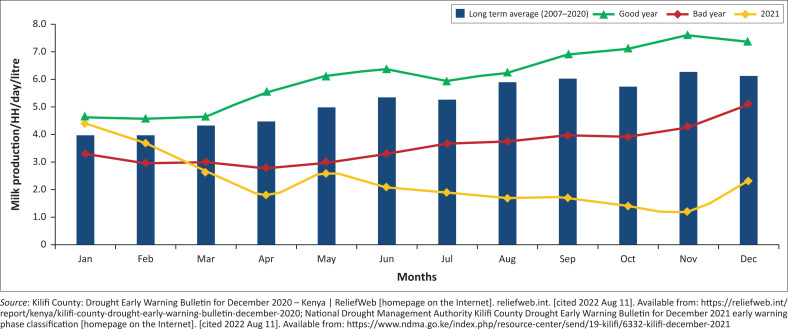
Average household milk production in Kilifi County.

The effects of a change in rainfall patterns were best demonstrated by reduced average household milk production in 2021; see Figure 2.^14^

### Impact of climate change: Community and health care workers’ experiences

Three themes arose from the FGDs: (1) lack of food variety, (2) the collapse of drought mitigating projects and (3) increasing poverty levels.

**Lack of food variety:** Inadequate and unpredictable rainfall has caused food scarcity and a lack of food variety in many households. Food consumption is currently based on eating ‘filling’ foods for survival rather than a consideration for a balanced diet:

‘[…*T*]here’s someone who may get 100 shillings [*USD 1*] and prefer to buy maize flour and eat *ugali* with salty water, rather than to buy vegetables and miss the flour.’ (FGD 5, 40-year-old, woman)

Insufficient homegrown vegetables have led to an increase in food prices:

‘Beans, spinach, cabbage, *mchicha* [*amaranth*], all these vegetables are sold, and you may not eat them because you don’t have money. I would need to buy cabbage worth 200 shillings [*USD 2*] (in a day).’ (FGD 5, 38-year-old, woman)

Attempts at the introduction of alternative sources of food to increase food variety in the face of harsh climatic conditions – for example, drought-resistant crops and chicken farming – have been met with much resistance, partly due to cultural concerns. For example, chicken is bred to reproduce and be sold, rather than to supplement nutrition in the household:

‘Your husband will not allow you to eat the eggs from chicken.’ (FGD 1, 19-year-old, woman)

To overcome this cultural barrier, CHVs felt that male involvement would help:

‘We need direct male involvement because nutrition is a family issue. But you’ll find that a lot of the time this information is being given to mothers only … because the man didn’t actually hear you saying it, they’ll not take it seriously.’ (FGD 6, 43-year-old, man)

Community health volunteers and HCWs have attempted to address food variety and adequate nutrition using community talks with picture charts of the various food groups. However, they felt that there is still a need to offer practical solutions that are in touch with the community needs, climate variability and available foods:

‘There’s talking and implementing, which are so different … currently, we just give talks on what is probably not available so the mother is left to go look for that food … they don’t implement what we’ve talked about.’ (FGD 6, 43-year-old, man)

**The collapse of drought-mitigating projects:** As a solution to the inevitable effects of climate change, especially in drought-prone areas, various projects have been initiated by nongovernmental organisations in recent years to mitigate food insecurity. These include kitchen vegetable gardens and chicken farming. Nevertheless, current unpredictable weather patterns have led to the drying up of vegetable gardens, rendering the project unsustainable:

‘We plant, but sometimes even the little water for washing dishes can get some other use because of the dry season … The vegetables will then dry up because of the lack of the water…’ (FGD 3, 18-year-old, woman).

Reduced vegetation cover has meant that livestock and humans compete for the few plants within the vegetable gardens. ‘[…Y]ou can plant and then you get a journey; by the time you’re back the goats have eaten the plants’ (FGD 3, 18-year-old woman). Chicken farming has also been hindered by diseases that may be contributed by changes in weather patterns:

‘We know how to rear chicken, but one thing fails us – the chicken disease … when the chicken get this disease, we don’t know the cure. All the chicken eventually die.’ (FGD 7, 52-year-old, woman)

Finally, inadequate support from the governmental bodies has threatened the sustainability of community projects that seek to address the impact of climate change on nutrition security:

‘We usually move with support, but sometimes we don’t have that governmental support since our facility is small…’ (FGD 8, 35-year-old, man)

**Increasing poverty levels:** Many residents rely on casual work for an income, for example, as a farmhand. Changes in rainfall patterns have translated into delayed farming or complete abandonment of farming practices for alternative income sources, reducing the income of other families who rely on casual farm work. Scarcity of cash then worsens undernutrition by reducing access to already difficult-to-get food supplies:

‘It’s been a dry season, we have no work; if you get today, tomorrow you don’t have.’ (FGD 4, 54-year-old, woman)

‘I can see the food is available in the market, but I have no fare to get there.’ (FGD 4, 45-year-old, woman)

## Discussion

This study reviewed both quantitative and qualitative data to assess the impact of climate change on drought and food availability on the community in Tsangatsini, Kilifi County. Kilifi County has one of the highest rates of undernutrition in Kenya. Local data from Tsangatsini Health Centre indicated that, in 2019, 23% of children seen between the age of 6 – 23 months were underweight. According to a 2016 nutritional assessment survey of Kilifi County, only 16% of households consume more than five food groups a day, and 39% of households are food-insecure.^7^ In a community with such few food reservoirs, unpredictable rainfall patterns have an immediate effect on the availability of food, posing a direct threat to the population’s nutrition status.

The community’s experiences demonstrated difficulty in accessing food due to experienced drought and challenges with continuing agricultural projects without support. This study emphasised the need to offer practical solutions to increase food availability and dietary diversity and to continuously educate and monitor behaviour change while overcoming cultural barriers. A COPC set-up can form a good platform to initiate this: residents in this study set-up worked with the community to prepare a meal plan based on affordable and available foods, and they proceeded to do a practical cooking class, preparing a multi-ingredient, low-cost, nutrient-rich porridge with the participation of mothers in the community. Although men were invited to the cooking class, only a few participated. Hence, more efforts have to be put into mobilising men to partake in such initiatives or at least in discussing the benefits of dietary change.

Lastly, county and government support is crucial to mitigate the effects of climate change on the nutrition of the community, by identifying opportunities for the utilisation of physical, financial and human assets.^16^ At the community level, physical assets may include putting up water-trapping tanks and protected water reservoirs; financial assets include household and community wealth creation and income diversification; human assets include embracing technology, distributing agricultural extension workers and educating and supporting farmers to embrace novel agricultural practices like embracing drought-resistant crops, drip irrigation and terrace planting.
